# Determination of Cefoperazone Sodium in Presence of Related Impurities by Linear Support Vector Regression and Partial Least Squares Chemometric Models

**DOI:** 10.1155/2015/593892

**Published:** 2015-11-19

**Authors:** Ibrahim A. Naguib, Eglal A. Abdelaleem, Hala E. Zaazaa, Essraa A. Hussein

**Affiliations:** ^1^Pharmaceutical Chemistry Department, Faculty of Pharmacy, University of Tabuk, Tabuk 71491, Saudi Arabia; ^2^Pharmaceutical Analytical Chemistry Department, Faculty of Pharmacy, Beni-Suef University, Alshaheed Shehata Ahmad Hegazy Street, Beni-Suef 62514, Egypt; ^3^Analytical Chemistry Department, Faculty of Pharmacy, Cairo University, Kasr El-Aini, Cairo 11562, Egypt

## Abstract

A comparison between partial least squares regression and support vector regression chemometric models is introduced in this study. The two models are implemented to analyze cefoperazone sodium in presence of its reported impurities, 7-aminocephalosporanic acid and 5-mercapto-1-methyl-tetrazole, in pure powders and in pharmaceutical formulations through processing UV spectroscopic data. For best results, a 3-factor 4-level experimental design was used, resulting in a training set of 16 mixtures containing different ratios of interfering moieties. For method validation, an independent test set consisting of 9 mixtures was used to test predictive ability of established models. The introduced results show the capability of the two proposed models to analyze cefoperazone in presence of its impurities 7-aminocephalosporanic acid and 5-mercapto-1-methyl-tetrazole with high trueness and selectivity (101.87 ± 0.708 and 101.43 ± 0.536 for PLSR and linear SVR, resp.). Analysis results of drug products were statistically compared to a reported HPLC method showing no significant difference in trueness and precision, indicating the capability of the suggested multivariate calibration models to be reliable and adequate for routine quality control analysis of drug product. SVR offers more accurate results with lower prediction error compared to PLSR model; however, PLSR is easy to handle and fast to optimize.

## 1. Introduction

**Figure 1:**
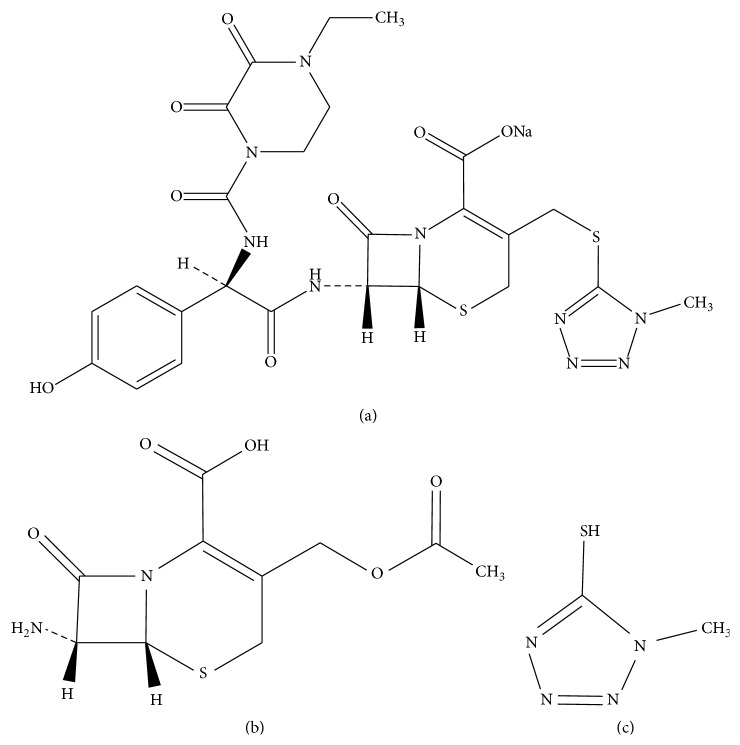
The chemical structure of CEF (a) and its reported impurities 7-ACA (b) and 5-MER (c).

Cefoperazone sodium (CEF), (6R,7R)-7-[[(2R)-2-[[(4-ethyl-2,3-dioxopiperazin-1-yl)carbonyl]amino]-2-(4-hydroxyphenyl)acetyl]amino]-3-[[(1-methyl-1H-tetrazol-5-yl) sulphanyl]methyl]-8-oxo-5-thia-1-azabicyclo[4.2.0]oct-2-ene-2-carboxylate (Figure 1(a)) [1], is a third-generation cephalosporin that acts by inhibiting bacterial cell wall biosynthesis [2]. According to B. P. [3], 7-aminocephalosporanic acid (7-ACA) (Figure 1(b)), (6*R*,7*R*)-3-[(acetyloxy)methyl]-7-amino-8-oxo-5-thia-1-azabicyclo[4.2.0]oct-2-ene-2-carboxylic acid, and 5-mercapto-1-methyl-tetrazole (5-MER) (Figure 1(c)), 1-methyl-1H-tetrazole-5-thiol, are deemed as potential impurities for CEF. 7-ACA is a significant and key intermediate needed for the synthesis of semisynthetic cephalosporin antibiotics in pharmaceutical industries [4]. Additionally, 7-ACA is the core chemical structure of cephalosporins. Chemical compounds containing this core are known to be relatively stable to hydrolysis and tolerant to *β*-lactamase, the enzyme that hydrolyzes cephalosporins. Accordingly, 7-ACA is a very important moiety for the activity of all cephalosporins. 5-MER is one of tetrazole derivatives that are used as intermediate of cephalosporin side chains.

Literature review presents several analytical methods for assay of CEF in its pharmaceutical formulation including spectrophotometric methods for determination of CEF [5, 6], near-infrared reflectance spectroscopy [7], and derivative UV spectrophotometry for determination of CEF in combination with sulbactam [8]. Chromatographic methods were applied for analysis of CEF and sulbactam [9, 10]; besides, an HPLC method with *β*-cyclodextrin stationary phase for determination of CEF, ampicillin, and sulbactam was reported [11]. CEF and sulbactam were determined in plasma also by LC-MS/MS method [12]. Additionally, electrochemical behavior and voltammetric determination of CEF [13, 14] were reported.

There are two main aims for the presented study. Firstly, the presented chemometric models show the ability of multivariate models to analyze selectively CEF in ternary mixtures with its two reported impurities using cost-effective and available instruments like UV spectrophotometer. Second, the presented study aims to compare two methods of multivariate calibration, PLSR and linear SVR chemometric models, through assay of CEF, 7-ACA, and 5-MER mixtures indicating the advantages and limitations of each model. The selected models offer better trueness and precision for quantitative determination of CEF in pharmaceutical formulation compared to the reported HPLC method [15].

## 2. Experimental

### 2.1. Instruments

A double beam UV-VIS spectrophotometer (SHIMADZU, Japan) model UV-1601 PC equipped with a quartz cell of 1 cm width and connected to IBM compatible computer was used. The software used was UVPC personal spectroscopy software version 3.7.

### 2.2. Material and Reagents

#### 2.2.1. Pure Standard

CEF was provided by Pharco Pharmaceuticals Co., Egypt. Both 7-ACA (CAS number 957-68-6) and 5-MER (CAS number 13183-79-4) were bought from Sigma-Aldrich through the Egyptian International Center for import and export (EIC, Egypt).

#### 2.2.2. Pharmaceutical Formulation

Cefobid 0.5 gm vials (batch number (2203)) were produced by Pfizer Pharmaceutical Industries Co. and cefoperazone 1 gm vials (batch number (1240395)) were manufactured by Sigmatec Pharmaceutical Industries Co. There are 3 concentrations available in the market for vials: 0.5 gm, 1 gm, and 1.5 gm.

#### 2.2.3. Chemicals and Reagents

All chemicals and solvents used in this study were of analytical grade, including methanol (E. Merck, Germany), water for injection B. P. 2003 (Egypt Otsuka Pharmaceutical Co., S.A.E., 10th of Ramadan City, ARE), and dipotassium hydrogen phosphate (K_2_HPO_4_) (El-Nasr Pharmaceutical Chemicals Co., Abu-Zaabal, Cairo, Egypt).

#### 2.2.4. Standard Solutions

(a) Stock standard solutions of 1 mg mL^−1^ for each of CEF, 7-ACA, and 5-MER were prepared in 3 mL of 0.05 M K_2_HPO_4_ solution and volume was completed with pure methanol. 7-ACA is soluble only in slightly alkaline solvent; accordingly the solubility was achieved by addition of fixed small volume of 0.05 M K_2_HPO_4_ solution before adding methanol. This step was carried out with all stock solutions of CEF, 7-ACA, 5-MER, and pharmaceutical formulation as well.

(b) Working standard solution of 100 *μ*g mL^−1^ of CEF was prepared in methanol. Two working standard solutions were prepared for each impurity. Working standard solution (1) of 100 *μ*g mL^−1^ and working standard solution (2) of 10 *μ*g mL^−1^ for both 7-ACA and 5-MER were prepared to allow preparation of final mixtures with very small concentrations of the impurities, up to 3% of CEF calculated on molar basis.

### 2.3. Linearity

**Figure 2:**
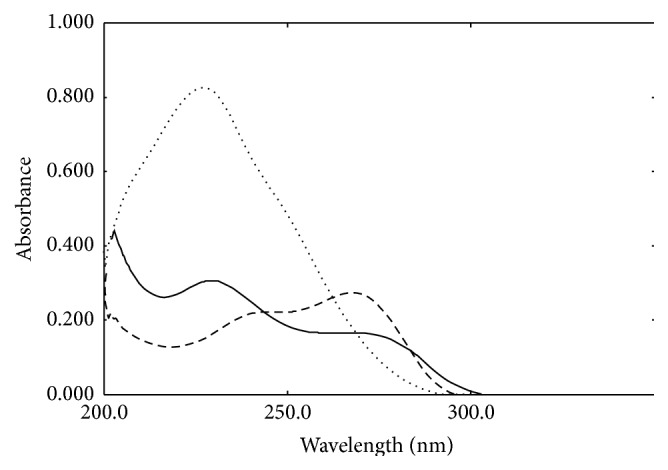
Zero order absorption spectra of 10 *μ*g mL^−1^ of CEF (—), 7-ACA (-  -  -), and 5-MER (…….) using methanol as blank.

UV spectra of a set of standards of CEF from 1 to 70 *μ*g mL^−1^ were recorded from 210 to 300 nm. CEF exhibited linearity between 5 and 50 *μ*g mL^−1^ at its *λ*_max_ is 229 nm. The superimposed spectra of 10 *μ*g mL^−1^ of CEF, 7-ACA, and 5-MER are shown in Figure 2.

### 2.4. Experimental Design

#### 2.4.1. Calibration Set

**Figure 3:**
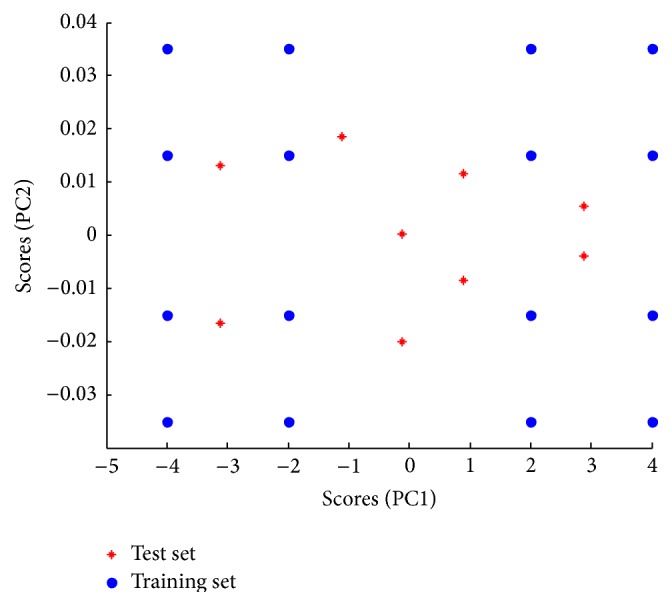
Scatter plot for scores of mean centered 16 training set samples and the 9 test set samples concentration matrices of the 4-level 3-component experimental design.

A 4-level, 3-factor calibration design was implemented using 4 concentration levels coded as +2, +1, −1, and +1, where (−1) is the central level for each of the components to be analyzed including the main drug (CEF) and its two impurities (7-ACA and 5-MER). The design aims to span the mixture space appropriately, where 4 mixtures for every compound at every concentration level exist, ending up with 16 mixtures for the training set [16]. The central level selected for the design was 20 *μ*g mL^−1^ for CEF and the concentration of each level for CEF depended on its calibration range. Concentration levels of impurities were based on involving impurities in an amount up to 3% of CEF calculated on molar basis to span most of the probabilities in future analyses.  Table 1 represents the design matrix for concentrations. 2D scores plot of the first two PCs of the concentration matrix was drawn to affirm the orthogonality, rotatability, and symmetry of the training set mixtures (presented as circles) as shown in Figure 3. Mean centering of all types of data was the best preprocessing method to provide best results.

#### 2.4.2. Test Set

To examine the validity and prediction capabilities of the introduced chemometric models, the independent test set mixtures were obtained by preparation of nine independent mixtures other than the training set mixtures but within the concentration space of the design as indicated in Table 1. The well position of the mixtures of both training set and test set mixtures is shown in Figure 3.

#### 2.4.3. Analysis of Cefobid and Cefoperazone Vials

For each of the two dosage forms, accurately weighed aliquot equivalent to 100 mg of CEF was transferred into 100 mL volumetric flask. To prepare stock solution, 3 mL of 0.05 M K_2_HPO_4_ solution was added and volume was completed using pure methanol. The solution was then diluted to prepare 100 *μ*g mL^−1^ working solution using methanol as solvent. Lastly, 2 mL portion of the working solution was diluted to 10 mL with methanol. The average of three corresponding spectra was recorded. This experiment was replicated six times and the produced spectra were analyzed by the suggested models.

### 2.5. Software

Codes for PLSR (PLS1 algorithm [17], bootstrap, and grid search were written using Matlab 7.5.0 (R2007b)). The codes for SVR algorithm were obtained from the internet website http://onlinesvr.altervista.org/.

## 3. Chemometric Methods

Multivariate calibration models are chemometric tools that set a relation between the spectra in data matrix **X** and the concentrations in data vector **c**. Multiple linear regression (MLR), principal component regression (PCR), and partial least squares regression (PLSR) are among the common models used for pharmaceutical analysis. PCR and PLSR methods are more preferable than MLR, because MLR needs further variable selection steps to perform optimally and to avoid multicollinearity. Additionally, PCR and PLSR can cope with a large number of spectral variables by decomposing the **X** data matrix into a relatively small number of scores. The scores matrix **T** then replaces the original **X** data matrix in the further steps. PLSR is more developed than PCR, where the **c** data vector shares in construction of the scores as well [17, 18]. Furthermore, the compression to a small number of scores works as a useful filter for noise [19]. Hence, PLSR is implemented in our presented analysis.

### 3.1. Partial Least Squares Regression (PLSR)

The PLSR model depends on the theory of existence of a linear relation between the **X** data matrix and the independent variables in concentration vector **c** [20]. The data matrix **X** and the response vector **c** are decomposed using a given number of PLS components (latent variable LVs) [21–24] as follows:

(1) X=T·P+E,c=T·q+f,

where **T** and **P** are the scores and loadings for **X** and **q** is the loading vector for **c**. **E** and **f** are the residuals for **X** and **c**, respectively. PLSR is considered one of the best in multivariate calibration because it is reported to perform better than MLR and PCR in several pharmaceutical applications [20].

*Optimization of Number of PLS Components for PLSR Model.* For prediction of optimum number of PLS components, bootstrap technique [25, 26] was used. This technique is based on dividing the original training set to two-thirds (bootstrap training set) and one-third (bootstrap test set). The PLSR model is then applied through building a model with the bootstrap training set to predict concentrations in the bootstrap test set and calculating the prediction error through the following equation:

(2) $$\begin{array}{c} RMSEP=\sqrt{\frac{1}{N}\overset{N}{\underset{n=1}{\sum }}{\left(c_{n}-{\hat{c}}_{n}^{A}\right)}^{2}}, \end{array}$$

where *N* is the number of bootstrap test set samples, *c*_*n*_ is the known concentration of sample *n*, and ${\hat{c}}_{n}^{A}$ is the corresponding predicted concentration at the defined number of PLS components. Equation (2) represents just one iteration out of 1000 iterations that were implemented in the presented study. The higher the number of iterations, the higher the probability of selecting all samples in both data sets (training set and test set). Finally a plot was established of the average of the 1000 root mean square error of prediction (RMSEP) values for different number of PLS components against the corresponding number of PLS components to choose the optimum number of optimum PLS components. The bootstrap training set was mean centered with every iteration.

### 3.2. Support Vector Regression (SVR)

For a data set **X**  (*I* × *J*) of an output vector **c**, SVR models aim to find a multivariate regression function *f*(*x*) that depends on **X** to predict a desired response (e.g., concentration of a chemical compound) from an object (e.g., a spectrum). SVR model equations are illustrated in literature [26–28] and the summary equation can be given as follows:

(3) $$\begin{array}{c} f\left(\;x\;\right)=\overset{N}{\underset{i,j=1}{\sum }}\left(\;\alpha_{i}-\alpha_{i}^{*}\;\right)\langle \;\emptyset \left(\;x_{i}\;\right)\cdot \emptyset \left(\;x_{i}\;\right)\;\rangle +b, \end{array}$$

where *α*_*i*_ and *α*_*i*_^*∗*^ are the Lagrange multipliers that fit to the constraint 0 ≤ *α*_*i*_, *α*_*i*_^*∗*^ ≤ *C*. *C* is known as penalty error or regularization constant which determines the trade-off between model simplicity and training error. The parameter *b* is the offset of regression function *f*(*x*). Further illustrations of (3) and the parameters *α* and *C* are found in literature [27, 29, 30]. *ϵ*-insensitive loss function is an additional factor commonly applied for SVR and will be used and optimized in this study [31, 32]. SVR method can be applied for both linear and nonlinear data. Linear SVR model is used in this study, where the used spectral data exhibit linearity guaranteed through the well planned experimental design.

Finally, in prediction step, unknown $\hat{c}$ value can be calculated as given below [33]:

(4) $$\begin{array}{c} \hat{c}=\overset{I}{\underset{i=1}{\sum }}\left(\;\alpha_{i}-\alpha_{i}^{*}\;\right)xx_{j}+b. \end{array}$$

*Optimization of Number of the Linear SVR Model Parameters.* Optimum *ϵ* and *C* values were calculated by using a grid search that depends on 4-fold cross validation to give the lowest root mean square error of cross validation (RMSECV). Primary range of values was set for *ϵ* (0.01–1) and *C* (30–1000). For each set of SVR parameters, 4 samples (*N* = 4) were taken out; the linear established SVR model was applied on the remaining 12 (*I* − *N*) samples. Further, RMSECV was predicted for the *N* samples and finally, the average of RMSECV after removal of all samples was calculated as follows:

(5) $$\begin{array}{c} RMSECV=\sqrt{\frac{1}{I}\overset{I}{\underset{i=1}{\sum }}{\left(c_{i}-{\hat{c}}_{i}\right)}^{2}}, \end{array}$$

where *c*_*i*_ is the true concentration for sample *n* and ${\hat{c}}_{i}$ is the corresponding predicted concentration.

## 4. Results and Discussion

### 4.1. Parameters' Optimization Results

**Figure 4:**
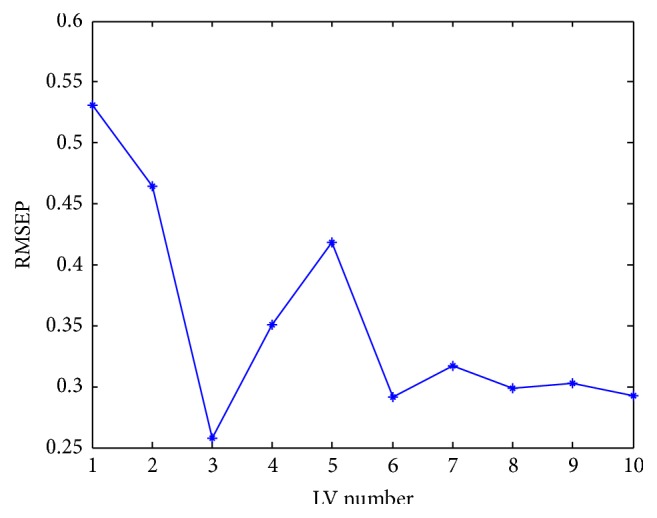
Choice of optimum number of PLS components (latent variables (LVs)) through plotting number of PLS components against the corresponding root mean square error of prediction (RMSEP) by using the bootstrap method.

Bootstrap technique was applied to choose optimum number of PLS components to build the best calibration model based on the training set. The optimum number was “three” as shown in Figure 4. Concerning SVR, the grid search that gave the lowest RMSECV (5) resulted in the values (*ϵ* = 0.02 and *C* = 280).

### 4.2. Data Analysis Results

**Figure 5:**
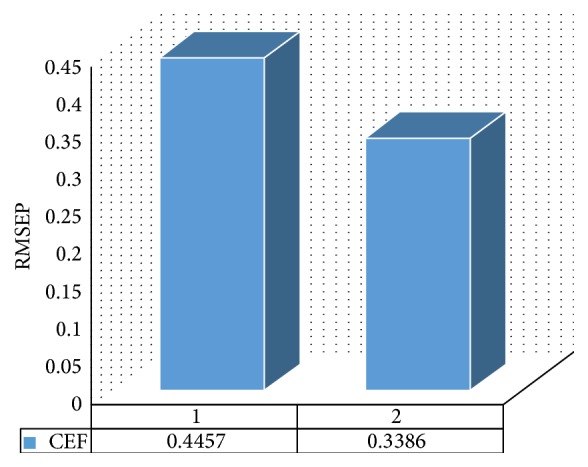
RMSEP bar plots for prediction of independent test set samples for CEF using 1: PLSR and 2: linear SVR models.

This study aims to introduce a comparative study between two chemometric methods known as PLSR and linear SVR via analysis of CEF in presence of its reported impurities: 7-ACA and 5-MER. The two multivariate models could handle the UV data and overcome the overlapping difficulty of the components' spectra shown in Figure 2. Both models were successfully able to determine the concentrations of CEF in training set and test set manifested by high recovery % with low SD as presented in Table 2. The RMSEP is a parameter used to evaluate the prediction abilities of the two models, Table 2. RMSEP comparative plot between PLSR and linear SVR for prediction of test set samples is presented in Figure 5.

There are many ways of comparison included in our study such as root mean square error of calibration (RMSEC), root mean square error of prediction (RMSEP), and calculation intensity and optimization steps. First, it was observed that linear SVR gives the least RMSEC (0.1960) compared to PLSR (0.2306) indicating better trueness and the corresponding standard deviation is smaller also indicating better precision. Second, the comparative bar plot in Figure 5 indicates that linear SVR gives the least RMSEP (0.3386) compared to PLSR (0.4457) indicating higher ability to process future samples and better generalization ability of linear SVR when compared to PLSR.

Concerning calculation intensity, PLSR is computationally simpler than SVR which is deemed to be more intense in calculation and time-consuming because of intense calculations for optimization. Choice of optimum parameters' values for SVR model could be misleading, where parameters that give the lowest RMSECV can be used (considered as overfitting as in PLSR) but still give better RMSEP or vice versa. In this study, we apply 4-fold cross validation to optimize SVR parameters to evade overfitting through prediction of small subsets of data instead of single sample (as used in leave-one-out cross validation technique), so improving the robustness of model and its generalization ability. The model that is more subject to overfitting is usually less robust. Accordingly, with PLSR, selecting too many components will lead to less robust model, that is, with less ability to predict future samples that have unknown signals. SVR parameters are calculated by using *k*-fold cross validation to evade overfitting, showing better robustness and higher prediction ability for future samples and hence being a more general model [34]. The probability of adherence of SVR to overfitting is less common than PLSR [35, 36]. The implemented linear SVR model in this study has another merit of optimizing only two parameters, unlike the nonlinear SVR models that use kernels and need optimization of more parameters and hence are more time-consuming in optimization process.

In conclusion, PLSR model is considered as one of the best in multivariate calibration and usually used in quality control routine applications and industry. It proved to be simple in optimization and computation and gives comparable results to reference HPLC methods in spite of processing simple UV data. However, SVR is still considered as a more general model with higher predictive ability for future samples.

### 4.3. Application of the Proposed Methods to the Pharmaceutical Formulation

The proposed chemometric methods were implemented for analysis of CEF in Cefobid 0.5 gr vial and cefoperazone 1 gr vial and satisfactory results with good recoveries were obtained. These results were statistically compared to the results obtained by applying the reported HPLC method [15] using *t*- and *F*-tests. The calculated *t*- and *F*-values are less than the tabulated ones showing no significant difference between the two introduced models and the reference HPLC method with regarding both trueness and precision, Table 3.

## 5. Conclusion

In general, the goals of this paper are presenting two multivariate chemometric models, PLSR and linear SVR, for analysis of CEF in presence of its reported impurities and making a modest comparison between the two models highlighting the advantages and limitations of each. Concerning predictive ability, the linear SVR proved to be better than PLSR according to RMSEP values indicating better generalization ability. However, PLSR is simpler and fast to optimize.

The two chemometric methods were also applied for the pharmaceutical formulations and statistically compared to reference HPLC method [15]. The calculated *t*- and *F*-values were found to be less than tabulated ones showing no significant difference in respect to both trueness and precision. The proposed methods showed high selectivity and trueness. The presented advantages of the proposed models suggest their use for routine quality control analysis without interference of normally encountered pharmaceutical additives or impurities that could be present in minor ratios.

Additionally, the obtained results affirm the possibility of using modern chemometric approaches, especially linear SVR, for assay of different pharmaceutical dosage forms using accessible cheap and simple instruments like UV spectrophotometer even in presence of large number of interfering components with extremely overlapped spectra.

## Figures and Tables

**Table 1:** Concentration design matrices in *µ*g mL^−1^ for the 4-level 3-factor experimental design, showing 16 training set mixtures together with the 9 test set mixtures.

Training set			Test set		
CEF	7-ACA	5-MER	CEF	7-ACA	5-MER
18	0.13	0.065	25	0.17	0.09
18	0.15	0.07	19	0.14	0.07
20	0.15	0.09	22	0.18	0.075
20	0.2	0.07	21	0.14	0.08
26	0.15	0.065	23	0.15	0.065
20	0.13	0.085	25	0.2	0.085
18	0.18	0.085	19	0.13	0.08
24	0.18	0.07	22	0.16	0.07
24	0.15	0.085	21	0.16	0.08
20	0.18	0.065			
24	0.13	0.09			
18	0.2	0.09			
26	0.2	0.085			
26	0.18	0.09			
24	0.2	0.065			
26	0.13	0.07			

**Table 2:** Assay results for prediction of training set (autoprediction) and independent test set of CEF by PLSR and linear SVR chemometric models.

Training set	PLSR		Linear SVR	
Taken (*µ*g mL^−1^)	Found (*µ*g mL^−1^)	*R*%	Found (*µ*g mL^−1^)	*R*%
18	18.577	103.21	18.629	103.50
18	18.029	100.16	18.136	100.76
20	19.850	99.25	19.920	99.60
20	19.889	99.44	19.980	99.90
26	26.114	100.44	26.110	100.42
20	19.899	99.49	19.971	99.85
18	17.897	99.43	17.980	99.89
24	23.737	98.90	23.754	98.97
24	23.997	99.99	23.980	99.92
20	19.727	98.63	19.775	98.87
24	24.005	100.02	23.980	99.92
18	18.089	100.50	18.116	100.65
26	26.236	100.91	26.143	100.55
26	25.919	99.69	25.980	99.92
24	24.118	100.49	24.181	100.76
26	25.917	99.68	25.980	99.92
Mean (%)		**100.01**		**100.21**
SD		**1.050**		**1.036**
RMSEC		**0.2306**		**0.1960**

Test set	PLSR		Linear SVR	
Taken (*µ*g mL^−1^)	Found (*µ*g mL^−1^)	*R*%	Found (*µ*g mL^−1^)	*R*%

25	25.443	101.77	25.320	101.28
19	19.069	100.36	19.067	100.35
22	22.357	101.62	22.278	101.27
21	21.403	101.92	21.330	101.57
23	23.515	102.24	23.397	101.73
25	25.630	102.52	25.440	101.76
19	19.294	101.55	19.238	101.25
22	22.432	101.96	22.296	101.35
21	21.603	102.87	21.493	102.35
Mean (%)		**101.87**		**101.43**
SD		**0.7082**		**0.5359**
RMSEP		**0.4457**		**0.3386**

**Table:** (a) Cefobid vial

Parameters	PLSR	Linear SVR	Reported HPLC method^*∗∗*^
Mean	102.77	100.30	102.85
SD	0.898	1.031	1.424
Variance	0.807	1.063	2.027
*n*	6	6	6
Student's *t*-test^*∗*^ (2.228)	0.907	0.006	—
*F*-test^*∗*^ (5.050)	2.513	1.907	—

**Table:** (b) Cefoperazone vial

Parameters	PLSR	Linear SVR	Reported HPLC method^*∗∗*^
Mean	98.17	99.33	99.40
SD	1.078	0.993	1.317
Variance	1.161	0.985	1.736
*n*	6	6	6
Student's *t*-test^*∗*^ (2.228)	0.108	0.914	—
*F*-test^*∗*^ (5.050)	1.223	1.327	—

^*∗*^The values between parenthesis are corresponding to the theoretical values of *t* and *F* (*P* = 0.05).

^*∗∗*^Reference method is HPLC [15].
